# RealPrice: Blockchain-Powered Real-Time Pricing for Software-Defined Enabled Edge Network

**DOI:** 10.3390/s22249639

**Published:** 2022-12-08

**Authors:** Yustus Eko Oktian, Thi-Thu-Huong Le, Uk Jo, Howon Kim

**Affiliations:** 1 Blockchain Platform Research Center, Pusan National University, Busan 609735, Republic of Korea; 2 IoT Research Center, Pusan National University, Busan 609735, Republic of Korea; 3 School of Computer Science and Engineering, Pusan National University, Busan 609735, Republic of Korea

**Keywords:** real-time pricing, software-defined network, blockchain, smart contract

## Abstract

With the limited Internet bandwidth in a given area, unlimited data plans can create congestion because there is no retribution for transmitting many packets. The real-time pricing mechanism can inform users of their Internet consumption to limit congestion during peak hours. However, implementing real-time pricing is opex-heavy from the network provider side and requires high-integrity operations to gain consumer trust. This paper aims to leverage the software-defined network to solve the opex issues and blockchain technology to solve trust issues. First, the network congestion level in a given area is analyzed. Then, the price is adjusted accordingly. Devices that send a lot of traffic during congestion will be charged more expensive bills than if transmitting traffic during an off-peak period. To prevent over-charging, the consumers can pre-configure a customized Internet profile stating how many data bytes they are willing to send during congestion. The software-defined controller also authenticates consumers and checks whether they have enough token deposits in the blockchain as Internet usage fees. We implement our work using Ethereum and POX controllers. The experiment results show that the proposed real-time pricing can be performed seamlessly, and the network provider can reap up to 72.91% more profits than existing approaches, such as usage-based pricing or time-dependent pricing. The fairness and trustability of real-time pricing is also guaranteed through the proof-of-usage mechanism and the transparency of the blockchain.

## 1. Introduction

Internet Service Providers (ISP) have historically applied a flat-rate pricing (FRP) mechanism, where consumers pay a monthly bill for a specific amount of bandwidth capacity in the network. However, as Internet usage becomes more popular and varied,  bandwidth becomes more and more limited. Network congestion then often happens as a consequence.

To reduce congestion, the ISP shifted its pricing strategy from FRP to usage-based pricing (UBP) [1], where consumers will be charged based on how much Internet they use. The intention is to make users aware of their network consumption so that they will wisely use the network, potentially reduce traffic and alleviate congestion. However, users in UBP are still expected to spend most of their bandwidth during peak periods, creating congestion only during that specific period. To further reduce congestion, the ISP offers time-dependent pricing (TDP) [2] and real-time pricing (RTP) [3], where the ISP adjusts the UBP price based on the congestion level of the network. The price gets more expensive when the traffic is congested and cheaper when the network is under-utilized.

In terms of the effectiveness in slowing down traffic congestion, we hypothesize that the RTP approach is the best compared to the alternatives because RTP can react to the dynamic of the traffic, providing the most accurate representation of the network state. However, performing RTP is sophisticated as it requires a feedback-loop mechanism [3]. The ISP must use current network information and determine the congestion level. After that, the ISP applies the updated price based on the detected level. This procedure must be repeated periodically 24/7. Unless we can automate this process and reduce opex, performing RTP becomes an unnecessary burden from the ISP side [4].

On the other hand,  consumers may not trust ISP operations because of their heavily centralized control [5]. In particular, malicious ISPs can tamper with the Internet usage statistics from users to charge consumers more UBP bills than it is supposed to. The ISP can also deny Internet access to users even though the users are eligible for access. This may impose an untrusted relationship between consumers and ISPs [6], which economically hurts the ISP if this results in losing consumers. Thus, implementing RTP without a trust guarantee from consumers is a risk for ISP.

Our research is targeted at filling in this important gap by combining the concept of software-defined network (SDN) [7] and blockchain [8] technology to create an automated and trustable RTP system. To our knowledge, only a few studies have discussed the feasibility of RTP systems (e.g., [3]), while other RTP studies focus on the theoretical aspects (e.g., [9,10]). Still, a solution to solve RTP issues of reducing opex (through automated processing) and increasing the trust towards the RTP system (through some kind of proof-of-provisioning) has not been addressed yet. This paper becomes a preliminary attempt toward those directions.

The rest of this paper is organized as follows. We discuss related works and preliminaries to our work in Section 2 and Section 3. After that, the inner workings of our proposal are thoroughly explained in Section 4. A feasibility analysis through quantitative and qualitative evaluations is investigated in Section 5. We then discuss the limitations and possible improvements for future works in Section 6. Finally, we conclude in Section 7.

## 2. Literature Review

This section discusses several related works, including the pricing plan for Internet broadband, SDN-based network monitoring strategy, and blockchain-based trusted platform.

### 2.1. Internet Data Pricing Strategy

Several types of pricing schemes for Internet bills exist in the literature, and we mainly discuss UBP, TDP, and RTP.

*Usage-Based Pricing*: In UBP, consumers are charged based on how much Internet they use (can be calculated per KB/MB/GB rate) [1]. When consumers do not apply for an Internet plan, the ISP typically applies this UBP scheme out of the box. Another variant of UBP includes combining UBP with the quota mechanism [11]. For example, users can subscribe to a 10 GB monthly plan. The ISP will check if the users have consumed the available quota. Only after the cap is breached will the ISP charge users using UBP. This quota scheme is generally priced cheaper than the pure UBP approach.

*Time-Dependent Pricing*: Similar to UBP, TDP consumers are still charged based on the traffic they consume. However, the ISP varies the prices based on the time when the users access the Internet [2]. Typically, the ISP wants to charge users more expensive bills during peak than off-peak periods. This way, the ISP can mitigate traffic congestion as we can expect fewer people are willing to use the Internet at rising prices. For example, the ISP can charge 0.001 USD per 100 KB from 12 a.m. to 6 a.m. (off-peak), 0.005 USD per 100 KB from 6 a.m. to 6 p.m. (normal), and 0.010 USD per 100 KB from 6 p.m. to 12 a.m. (peak). The ISP put this plan ahead of time so that the users know the price schedule beforehand.

*Real-Time Pricing*: RTP is a variant of TDP, where instead of using static pricing across the day, the ISP adjusts the price dynamically according to the current congestion or demand level in the network [9,10]. Thus, in some literature, this RTP is often also known as “dynamic TDP” [3]. Before starting the network, the ISP publishes a price plan throughout the day similar to TDP (the plan should be predicted based on day-to-day traffic history) [12]. Then, the ISP monitors the current network condition; if the ISP finds sudden traffic congestion, the ISP updates the price accordingly. Similarly, the ISP reduces the prices when it finds that the network is under-utilized.

### 2.2. Network State Monitoring Using SDN

A data plan strategy that requires continuous monitoring of users’ network data (e.g., UBP, TDP, and RTP) is expected to have high opex costs. Several SDN approaches can be used to automate this process.

Haxhibeqiri et al. [13] proposed a novel monitoring technique using in-band network telemetry (INT) tailored explicitly for IEEE 802.11 networks. The authors showed that the proof of concept INT implementation could be used to monitor real-life SDN-based wireless networks with good accuracy and low overhead. Yao et al. [14] presented a self-learning control for network monitoring in an SDN network called NetworkAI. They leverage reinforcement learning and INT to produce dynamic control policies and optimal monitoring decisions. FlowSpy [15] proposed a load-balancing network monitoring for SDN using P4 (Programming Protocol-Independent Packet Processors) language and reduced the interaction overhead between the data plane and control plane in SDN. Therefore, the monitoring system can be performed in a scalable manner.

The OpenFlow protocol [7] provides a network application programming interface (API) to collect flow-level statistics from the underlying SDN switches. Therefore, the SDN controller can use this API to learn about the state of the network periodically and automate the UBP, TDP, and RTP process, reducing opex.

### 2.3. Blockchain and Smart Contract as Trusted Platform

Blockchain has become popular due to the adoption of Bitcoin [8] as the world-first truly decentralized payment system that does not require third-party intervention. Users send arbitrary signed transactions to the blockchain platform, which the miners then aggregate into hash chains of blocks. The consensus algorithm, for example, Proof-of-work (PoW) [8] or Practical Byzantine Fault Tolerance (PBFT) [16], replicates blocks to all miners, creating a distributed tamper-proof ledger.

The initial blockchain applications are limited only to cryptocurrencies. However, with the invention of smart contracts [17] through the Ethereum foundation, developers can now create decentralized applications (dapp) and further widen the blockchain applications outside of coins, for example, in supply-chain [18] and healthcare [19] areas. Most importantly,  smart contracts can be utilized as a trusted collaborated platform in an untrusted environment [20,21,22]. Similar to the previous studies, we leverage smart contracts as a trusted platform to run RTP operations such that the system can be audited and executed fairly for consumers and ISP.

## 3. Preliminaries

This section details the RTP trust and opex problems, which become the background and design rationale of the proposed RTP system.

### 3.1. Problem Statement

Due to the typically centralized governance of the ISP network, the feasibility and success of the RTP system depend on how much trust the consumers can give to the system. On the other hand, the consumers may also try to find holes and weaknesses to profit from the ISP. Thus, the RTP system may become a game-theory problem between the ISP and consumers [10], and we need to solve trust issues (T) from both sides.

**T1**  The consumers must know whether the Internet usage calculation has been performed correctly or not.

The Internet usage per user is typically calculated from the ISP side. Users may not trust such measurements because they are not involved in the measurement, and there is a lack of proof to validate the calculation.

**T2**  The ISP needs to know if the consumers have the money to pay the Internet bills before forwarding their traffic.

Ideally, the ISP must only allow well-funded consumers to access the network and reject users with no funds to prevent economic loss. However, the ISP may not know this information because there is no user balance verification procedure.

To solve the previously mentioned trust issues, the ISP must perform additional operations, which as a result, cause several opex problems (O) as follows.

**O1**  The RTP system is required to monitor the live traffic 24/7, which may result in heavy overhead.

A typical SDN-based monitoring method uses a pooling strategy, where the controller sends a FLOW-STATISTIC message to the switches periodically to query flow table states in the switches and then obtain the Internet usage per user from the flow rules. Depending on the query frequency, this approach may cause overhead because many packets must be transmitted between the controller and switch.

**O2**  The RTP system must provide verifiable proofs that the Internet usage measurement has been performed correctly.

The ISP needs to provide trustworthy and auditable proof of Internet usage. The proof must be generated by a trusted third party (not the ISP nor the consumer) and protected by a tamper-free mechanism to guarantee the proof’s integrity.

**O3**  The RTP system needs to authenticate the users and determine whether the consumers have money to pay for the Internet bills.

The user authentication and balance verification must be automated, accurate, and produce low overhead. Given a 24/7 real-time system, the ISPs must determine how to verify users, when to authenticate them, and how frequently such authentication must be performed. More frequent checks generally result in more overhead.

### 3.2. Design Consideration

SDN should help automate the RTP system and reduce opex, while blockchain can improve the system’s trustworthiness. We devised the following design decisions by leveraging both technologies for our proposed RTP system.

*Domain-Based Architecture*: We define a “domain” as an area coverage of a single-edge SDN switch. Inside this domain, there will be our potential consumers. The size of the domain depends on the number of devices that this switch serves. Ideally, we should have multiple smaller domains instead of a few big domains. This domain type can also vary depending on the physical network (e.g., residential, enterprise, cellular, or IoT network). The proposed RTP system will manage the network on a per-domain basis.

*Timeslot-Based Microprocessing*: For easy management, we divide the time of the day into smaller time units that we call a “timeslot”. This timeslot can be adjusted freely; for example, 1 h, 30 min, or 15 min. The proposed RTP system will manage the network operation on a per-timeslot basis.

*Asynchronous Internet Usage Monitoring*: Instead of using the FLOW-STATISTIC pooling method, we leverage FLOW-REMOVED messages to get the users’ Internet usage information when the flow rules expire. As a result, we can reduce the communication overhead by half and solve **O1**. Recall that FLOW-REMOVED is an asynchronous message from switches to the controller, and unlike FLOW-STATISTIC, the controller does not need to initiate a request message to trigger it.

*Off-Chain OpenFlow Messages and On-Chain Data Interaction Log*: We leverage the OpenFlow messages generated from the edge SDN switches as our verifiable proofs to support the RTP operations. This way, the switch becomes the trusted entity that generates RTP proofs. Through the produced FLOW-REMOVED messages, trusted validators can check the users’ byte usages from the messages and compare them with the ones claimed by the ISP. Furthermore, through the PACKET-IN messages, validators can also check that the users are indeed within the domain and judge whether the ISP is correct in providing the FLOW-MOD messages that result in the given FLOW-REMOVED messages. The hash of those messages will be stored in the blockchain as a tamper-proof log. The blockchain is also used to store any other related interactions between the ISP and consumers regarding the RTP operations. Therefore, this design solves **T1** and **O2**.

*User Verification Before Installing Flow Rules*: Since our RTP system manages the network on a per-timeslot basis, we take this chance as a window to perform user verification. We leverage the smart contract to build our customized cryptocurrency tokens as payment methods in our RTP method. Users must first deposit some tokens to the smart contract before using the Internet. Then, when receiving PACKET-IN messages from users, the system will authenticate and make sure that the users’ have deposited enough funds before sending FLOW-MOD messages to forward users’ traffic. The flow rules will expire at the end of the timeslot, and users must be re-authenticated if they want to continue using the Internet in the subsequent timeslots. Thus, this design can potentially solve **T2** and **O3**.

## 4. RealPrice Framework

In this section, we present our proposed RTP framework named RealPrice. Specifically, we briefly summarize the overall workflow of our proposal, and we then elaborate on the detailed design of SDN applications and smart contracts. In the end, we compare our work with the existing works.

### 4.1. Framework Overview

**Figure 1:**
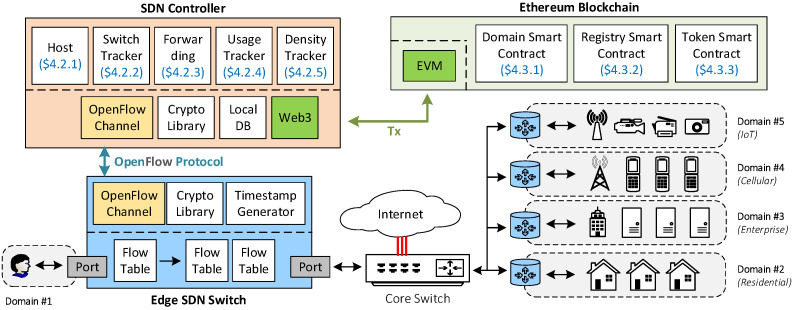
The system architecture of RealPrice consisting of SDN edge domains, SDN switches, SDN controllers, and the Ethereum blockchain network. RealPrice can be used in any edge network, such as residential, enterprise, cellular, or IoT networks.

Figure 1 depicts the overall architecture of RealPrice, and Table 1 describes important notations in this paper. Note that the information provided here is only the essential requirement. Because the actual implementation inside a given domain can vary from one case to another, developers can further tweak this framework when necessary. In each given timeslot, RealPrice has three objectives.

First, the system checks the current congestion level inside the domain. For a given interval, the SDN controller sends PORT-STATISTIC request messages to all underlying switches. The switches respond with PORT-STATISTIC reply messages, which contain information about the current transmitted and received bytes per port. From this statistic, the controller can calculate the current byte rate per port and determine if congestion happens in any given port. The controller then updates the congestion level accordingly.

Second, the system facilitates Internet routing for valid users. Consumers *u* (i.e., can be IoT gateways, IoT devices, PCs, laptops, smartphones, and modems) are previously registered in an off-chain database by recording their MAC address $\mu_{u}$ and in an on-chain database by saving their blockchain address $\alpha_{u}$. Consumers also add their customized Internet profiles, which are user-based policies to tell SDN controllers how to limit their traffic during congestion. During the network runtime, the PACKET-IN messages of traffics from well-funded registered users will be responded to with FLOW-MOD messages specifying the correct forwarding path and Internet profile based on the current congestion level in the given timeslot. Meanwhile, invalid PACKET-IN messages will be rejected, and non-registered users cannot access the network.

Third, the system records the consumers’ Internet usage in the previous timeslot via the FLOW-REMOVED messages. In those messages, we can find the byte count per user from the previously installed FLOW-MOD messages. The controller then calculates the Internet bills for each user based on the usage and density level in the given timeslot and confiscates the users’ deposit accordingly.

In the subsequent sections, we explain the inner working of our framework from both the SDN and the blockchain sides.

### 4.2. SDN Application Design

The SDN application is built using modular design, with each part playing a role in processing the network events from OpenFlow protocol [7] and responding to the events following the previously agreed rules stored in the database or smart contract.

#### 4.2.1. Host Module

The Host module manages users’ related information, such as blockchain address $\alpha_{u}$, MAC address $\mu_{u}$, and Internet profile. All this information is stored in the local database. Users can create a customized Internet profile, such as in Table 2. The Off profile indicates that the user does not perform any restriction on their bandwidth throughout any density level. The Moderate profile reduces the traffic by 25% in normal and 75% in peak. Meanwhile, the Aggressive profile cuts traffic by 50% in normal and drops any traffic during peak. Choosing the Off profile guarantees faster data transfer by paying more bills, especially when the transfer is made during congestion/peak. On the other hand, choosing the Aggressive profile is cost-efficient by compromising longer delays.

#### 4.2.2. Switch Tracker Module

This module leverages Link-Layer Discovery Protocol (LLDP) messages to determine whether a given switch’s port is considered an edge port (connected to the host) or not. Algorithm 1 summarizes the logic of the Switch Tracker module. During operations, switches will broadcast LLDP messages to their neighboring devices. If a switch receives LLDP messages in a given port, we can mark this port as a non-edge port. Meanwhile, ports not receiving LLDP messages are marked as edge ports. From this port configuration, we can determine edge switches. If a switch consists only of non-edge ports, we can confirm that this given switch is a non-edge switch. This module monitors the LLDP messages periodically and updates the edge/non-edge status of the switch over time via PORT-STATISTIC messages.
**Algorithm 1** The Switch Tracker module’s pseudocode.  1:**On** startup:  2:    **for all** p∈s **do**  3:        Mark *p* as edge port in database ▹ Port is set as edge by default  4:    Dispatch PORT-STATISTIC request messages every *x* s  5:**On** receiving LLDP messages:  6:    Get the switch id *s* and port number *p* from LLDP messages  7:    Mark *p* as non-edge port in database  8:**On** receiving PORT-STATISTIC reply messages:  9:    Get the switch id *s* and port number *p* from PORT-STATISTIC messages10:    **for all** p∈s **do**11:        **if** isEdgePort(s,p) **then** mark *s* as edge switch in database **return**12:    Mark *s* as non-edge switch in database

#### 4.2.3. Forwarding Module

The Forwarding module is responsible to handle inward and outward packets in the domain based on the previously agreed policy. Aside from that, the module must also perform user authentication during the PACKET-IN process so that only valid in-domain hosts can exist and use the network. Algorithm 2 summarizes our forwarding procedure.

**Part 1**  Host Authentication

Every time the controller receives PACKET-IN messages from edge switches, we check the MAC address of the incoming/outgoing port and perform authentication. First, we ensure that the host is on the list of registered users. Second, we ensure that the host has deposits so the controller can forward the sent packets without economic loss.

Since we do not know how much traffic this host will transfer in the next timeslot, we use an upper-bound deposit limit. We make sure that the user has money for maximum bandwidth transfers $\omega_{u}^{cap}$ in bit per second (bps) from the current timestamp $t_{now}$ to the next timeslot $T_{n+1}$. The $\rho_{n}$ is the data price rate in this timeslot $T_{n}$.
**Algorithm 2** The forwarding module’s pseudocode.  1:**On** startup:  2:    Configure the base value of PRIORITY tag  3:**On** receiving PACKET-IN messages:  4:    Get the switch id *s* and in-port number $p_{in}$ from PACKET-IN messages  5:    Get match information M from packet header:  6:        The source $\mu_{src}$ and destination $\mu_{dst}$ MAC address  7:        The source $\gamma_{src}$ and destination $\gamma_{dst}$ IP address  8:        The source $\theta_{src}$ and destination $\theta_{dst}$ TCP/UDP port number  9:    Get out-port number $p_{out}$ for M following the L2-SWITCH or L3-SWITCH algorithm10:    **if** isEdgeSwitch(s) **then**11:        **if** isEdgePort$\left(s,p_{in}\right)$ **then**               ▹ Upstream traffic12:           Set $\mu_{u}=\mu_{src}$13:           Set wildcard for $\mu_{dst}$14:        **else if** isEdgePort$\left(s,p_{out}\right)$ **then**           ▹ Downstream traffic15:           Set $\mu_{u}=\mu_{dst}$16:           Set wildcard for $\mu_{src}$17:        **if not** isRegisteredHost$\left(\mu_{u}\right)$ **then return**   ▹ Ignore non-registered users18:        Calculate required deposit $\delta =\rho_{n}\times \omega_{u}^{cap}\times \left(T_{n+1}-t_{now}\right)$19:        Get user balance $\beta_{u}\leftarrow$getHostBalance$\left(\mu_{u}\right)$20:        **if** $\beta_{u}<\delta$ **then return**    ▹ Ignore users because of insufficient balances21:        Set wildcard for $\gamma_{src},\gamma_{dst},\theta_{src},\theta_{dst}$22:        Set PRIORITY tag as base value $+(\left(T_{n}-T_{start}\right)/T_{window})$23:        Set HARD-TIMEOUT tag as $T_{n+1}-t_{now}$   ▹ Flow rules will expire at $T_{n+1}$24:        Get current density d←GetDensity$\left(T_{n}\right)$25:        Set QUEUE-ID to $\psi_{u}\leftarrow$GetUserProfile$\left(\mu_{u},d\right)$26:        Set FLOW-REMOVED tag to True27:    **else**28:        Set wildcard for $\mu_{src},\mu_{dst},\gamma_{src},\gamma_{dst},\theta_{src},\theta_{dst}$29:        Set priority using base value of PRIORITY tag30:        Set IDLE-TIMEOUT tag to 10 s31:        Set FLOW-REMOVED tag to False32:    Set match $M=\{p_{in},\mu_{src},\mu_{dst},\gamma_{src},\gamma_{dst},\theta_{src},\theta_{dst},p_{out}\}$33:    **if** FLOW-REMOVED = True **then** Save hashcode of M in database34:    Send FLOW-MOD messages with M to *s*

Packets from unregistered MAC addresses or packets tied to insufficient deposit balances will be dropped, and the controller will not create FLOW-MOD messages for such packets. Furthermore, we do not perform authentication for non-edge switches because we assume that non-edge switches are always connected to trusted non-host interfaces (e.g., switches, routers, servers, etc.). Nevertheless, such authentication can also be implemented in the future if needed.

**Part 2**  Flow Rules Installation

The forwarding path is calculated and determined following well-known forwarding algorithms. For example, using the conventional Layer2-learning (L2-SWITCH) and Layer3-learning switch (L3-SWITCH) algorithm, commonly present in typical SDN controllers. However, the forwarding rules are applied differently depending on whether we install the rules in the edge or non-edge switches.

We must track the users’ Internet consumption to charge them according to their usage. As a result, we need to install more granular match rules (in the L2 level) for users in the edge switches. We must use the no-wildcarded value in the destination MAC address field for inbound traffic. Conversely, when handling outbound traffic, we use a no-wildcarded value for the source MAC address. This way, we can track the device’s upstream and downstream usage information. On the other hand, the rules can be less granular for non-edge switches. We can use as many wildcards as possible in the match field as long as it provides basic packet forwarding to/from the adjacent switches.

The controller must also set the correct duration, priority, and queue-id for the flow rules. They must last only within the current timeslot. For this reason, we use HARD-TIMEOUT instead of IDLE-TIMEOUT to force the rules to expire when the timeslot ends. To mitigate the possibility of duplicate or conflicting flow rules, the priority number is set with respect to the start of the day $T_{start}$. Flow rules installed during the evening will have a higher priority number than those installed in the afternoon, and so on. Finally, the controller checks the current density for the given timeslot and applies a queue policy for users depending on their Internet profiles.

It is also worth noting that the FLOW-REMOVED tag must be set to True in the edge switch so that we can get the FLOW-REMOVED messages when these rules expire and capture the byte usage consumption. Meanwhile, since it is irrelevant, the FLOW-REMOVED tag can be set to False in the non-edge switch. To ensure that the controller can track all installed rules in the switches, we save the hashcode of match rules in the database before sending FLOW-MOD messages to the switches.

#### 4.2.4. Usage Tracker Module

The main goal of the Usage Tracker module is to capture the users’ byte usage during a given timeslot through the FLOW-REMOVED messages, which are generated by the previously installed FLOW-MOD messages from the forwarding module. Algorithm 3 summarizes our usage tracker procedure.
**Algorithm 3** The usage tracker module’s pseudocode.  1:// Flow rules are installed at $T_{n-1}$ and expires at $T_{n}$  2:**On** receiving FLOW-REMOVED messages:  3:    Get the switch id *s*, match rules M, byte count *B* and packet count *P*            from FLOW-REMOVED messages  4:    **if not** isEdgeSwitch(s) **then return**       ▹ Ignore non-edge switch  5:    **if** B≤0 **or** P≤0 **then return**           ▹ Ignore empty flow rules  6:    **if** hashcode of M∉ database **then return**  ▹ Ignore unknown flow rules  7:    Get $p_{in},\mu_{src},\mu_{dst}\leftarrow M$  8:    **if** isEdgePort$\left(s,p_{in}\right)$ **then**               ▹ Upstream traffic  9:        **if not** isRegisteredHost$\left(\mu_{src}\right)$ **then return** ▹ Ignore non-registered users10:        Add upload byte usage *B* of $\mu_{src}$ for $T_{n-1}$ in database11:    **else**                       ▹ Downstream traffic12:        **if not** isRegisteredHost$\left(\mu_{dst}\right)$ **then return** ▹ Ignore non-registered users13:        Add download byte usage *B* of $\mu_{dst}$ for $T_{n-1}$ in database14:    Delete hashcode of M from database

First, we need to filter the FLOW-REMOVED messages by performing several validations. Because users reside in edge switches, we ignore FLOW-REMOVED messages that come from non-edge switches. Furthermore, we also ignore expired flow rules that do not have any usage during their lifetime (packet count or byte count equals zero). Recall that in Algorithm 2, we store and track the hashcode of installed M flow rules in the database. Therefore, we can ignore FLOW-REMOVED messages that are not in our interest. Finally, we also set aside messages containing flow rules unrelated to our registered users.

Second, we capture the byte count metric *B* from FLOW-REMOVED messages and update the users’ upload or download usage accordingly. Note that we must update the usage for the previous timeslot $T_{n-1}$ because these messages correspond to the flow rules we installed during the previous timeslot. Once it is added to the database, we delete the hashcode of this match to prevent duplicate entries. Therefore, supposing that the controller receives two of the same FLOW-REMOVED messages, only the first one will be processed.

#### 4.2.5. Density Tracker Module

The density tracker module periodically sends and receives PORT-STATISTIC messages to analyze possible congestion in the domain. Algorithm 4 summarizes our congestion detection procedure.
**Algorithm 4** The density tracker module’s pseudocode.  1:**On** startup:  2:    Configure congestion level L, where $L=\{L_{1},L_{2},L_{3},...,L_{d},...,L_{D}\}$. *D* is the          total number of congestion level available. L contain softmax values          representing the distribution of total bandwidth threshold used in Q.  3:        $L_{1}=L_{offpeak}=0.5$; $L_{2}=L_{normal}=0.4$; $L_{3}=L_{peak}=0.1$  4:    Calculate the bandwidth threshold Q for each congestion level, where    $Q=\{Q_{1},Q_{2},Q_{3},\ldots ,Q_{d},\ldots ,Q_{D}\}$.  5:        $Q_{1}=L_{1}\times \omega_{domain}^{cap}$              ▹ 50% of domain’s bandwidth capacity  6:        $Q_{2}=\left(L_{1}+L_{2}\right)\times \omega_{domain}^{cap}$           ▹ 90% of domain’s bandwidth capacity  7:        $Q_{3}=\left(L_{1}+L_{2}+L_{3}\right)\times \omega_{domain}^{cap}$          ▹ 100% of domain’s bandwidth capacity  8:    Configure counters J for each congestion level, where $J=\{J_{1},J_{2},J_{3},\ldots ,J_{d},\ldots ,J_{D}\}$.  9:    Configure hit-count threshold *j* for J.10:        j=3            ▹ The congestion is updated whenever the counter reach 311:    Dispatch PORT-STATISTIC request messages every *x* s12:**On** receiving PORT-STATISTIC reply messages:13:    Get the switch id *s* and port number *p* from PORT-STATISTIC messages14:    **if not** isEdgeSwitch(s) **then return**              ▹ Ignore non-edge switch15:    **if** isEdgePort(s,p) **then return**                     ▹ Ignore edge port16:    Get the last byte count $r_{prev}$ and last timestamp $t_{prev}$ for *s* from database17:    Get the current byte count $r_{now}$ from PORT-STATISTIC messages18:    Calculate the current rate as $R=\frac{r_{now}-r_{prev}}{t_{now}-t_{prev}}$19:    Update $r_{prev}=r_{now}$ and $t_{prev}=t_{now}$ 20:    **if** $R\leq Q_{1}$ **then** $J_{1}\leftarrow J_{1}+1$                ▹ Increment off-peak counter21:    **else if** $R>Q_{1}$ **and** $R\leq Q_{2}$ **then** $J_{2}\leftarrow J_{2}+1$        ▹ Increment normal counter22:    **else if** $R>Q_{2}$ **and** $R\leq Q_{3}$ **then** $J_{3}\leftarrow J_{3}+1$         ▹ Increment peak counter23:    Get current density level *d* from database24:    triggered=False25:    **if** $J_{1}=j$ **and** d<1 **then**26:        Set d=1; triggered=True               ▹ Set current density as off-peak27:    **else if** $J_{2}=j$ **and** d<2       **then**28:        Set d=2; triggered=True                ▹ Set current density as normal29:    **else if** $J_{3}=j$ **and** d<3 **then**30:        Set d=3; triggered=True                 ▹ Set current density as peak31:    **if** triggered **then**32:        Reset counters $J_{1}=0$, $J_{2}=0$, and $J_{3}=0$33:        Create FLOW-MOD message to clear all flow rules in *s*34:**On** new timeslot $T_{n}$:35:    Create FLOW-MOD message to clear all flow rules in all edge switches

We are interested in the inward/outward traffic of our domain. Therefore, we monitor congestion on non-edge ports of edge switches in all domains and ignore irrelevant PORT-STATISTIC messages. We measure the current traffic rate by comparing the byte count information from the current PORT-STATISTIC $r_{now}$ with the last time we received PORT-STATISTIC $r_{prev}$. We then determine whether the rate *R* can be categorized as off-peak (d=1), normal (d=2), or peak rate (d=3), and increment the counter *J* accordingly.

To ensure the stability of congestion level within a given timeslot, we enforce three density rules, as follows:

**Rule** **1** The counter must be triggered multiple times before the system can change the current density level.

Because of the possibility of ephemeral burst/dropped traffic, we need to wait for the counter to be triggered for up to the hit-count threshold *j* parameter before changing the current density level in a domain. Without this waiting period, the system may misclassify traffic patterns.

**Rule** **2** Within a given timeslot, the density level can only move upward but not downward.

For example, an off-peak can become normal, and normal can become peak. However, normal cannot move back to off-peak, and peak density cannot return to normal or off-peak. Without enforcing **Rule 1** and **Rule 2**, the congestion may change many times within a timeslot. Numerous updates will not efficiently solve the congestion problem and will confuse consumers.

**Rule** **3** The density level is reset and replaced with a default value at the beginning of each timeslot.

This rule is applied to complement **Rule 2**. We periodically reset the density level to reduce the density level from the previous timeslot if the traffic slows down.

To change the density level, the controller first creates FLOW-MOD messages to clear/delete all flow rules in the affected switches. The subsequent packets from the domain will trigger new PACKET-IN messages, to which the controller responds by creating new flow rules with the updated density level *d* and queue id $\psi_{u}$ from the database. We perform this “deletion then insertion” strategy to ensure the safety and correctness of the switch state and eliminate possible race conditions or flow rules conflicts that may happen in the flow table. The deletion still triggers the FLOW-REMOVED messages; therefore, the consumers’ usage from the deleted flow rules can still be captured safely.

### 4.3. Smart Contract Design

Similar to SDN application designs, we design the smart contract to be modular as well by using a multi-contract environment. Algorithm 5 summarizes several essential methods in our smart contract.
**Algorithm 5** The smart contract pseudocode.  1:// For DSC, simple setter or getter methods to implement database operations in           Algorithms 1–4 are omitted here.  2:// TSC is assumed to use standard ERC20 token contract design.              So, we refrain from explaining the detail here.  3:**procedure**addDomain($\alpha_{DSC}$)                     ▹ On RSC  4:    **if** $\alpha_{sender}$ **not** contract owner **then return**      ▹ Can only be called by owner  5:    Save $\alpha_{DSC}$ in R, list of trusted domain contracts  6:**function**isRegisteredDomain($\alpha_{DSC}$)                  ▹ On RSC  7:    **if** $\alpha_{DSC}\in R$ **then return** True  8:    **else return** False  9:**procedure**deposit(token amount *f*)                   ▹ On DSC10:    **if** $\alpha_{sender}\notin U$, list of registered users **then return**        ▹ Only for user11:    Get balance $\beta_{u}$ from TSC using $\alpha_{sender}$12:    **if** $\beta_{u}<f$ **then return**                  ▹ Insufficient balance13:    Increase allowance and lock deposit *f* for $\alpha_{sender}$ in TSC14:**procedure**refund(*f*)                         ▹ On DSC15:    **if** $\alpha_{sender}\notin U$ **then return**            ▹ Can only be called by user16:    Decrease and unlock allowance of $\alpha_{sender}$ for *f* amount from TSC.17:**procedure**withdraw($\alpha_{u}$, $T_{n}$)                          ▹ On DSC18:    **if** $\alpha_{sender}\notin C$, list of registered controllers **then return**   ▹ Only for controller19:    **if** $t_{now}<T_{n}$ **then return**      ▹ Can only be called after the timeslot ends20:    Get usage proof *y* ← getProofsage$\left(\alpha_{u},T_{n}\right)$21:    **if** *y* **not** found **then return**       ▹ Must submit proof before withdrawal22:    Get density level *d* ← getDensity$\left(T_{n}\right)$23:    Get price rate $\rho_{n}$ at $T_{n}$ based on density *d*24:    Get upload $B_{u}^{up}$ and download $B_{u}^{down}$ usage ← getUsage$\left(\alpha_{u},T_{n}\right)$25:    Calculate bills $b=\left(\rho_{n}\times B_{u}^{up}\right)+\left(\rho_{n}\times B_{u}^{down}\right)$26:    Call tranferFrom method in TSC to withdraw *b* amount from $\alpha_{u}$27:**procedure**addProofUsage($\alpha_{u}$, $T_{n}$, *y*)                  ▹ On DSC28:    **if** $\alpha_{sender}\notin C$ **then return**            ▹ Can only be called by controller29:    **if** $t_{now}<T_{n}$**then return**        ▹ Can only be called after the timeslot ends30:    Add *y* at $T_{n}$ for $\alpha_{u}$ to Y, a list of stored proofs.

#### 4.3.1. Domain Smart Contract

Each domain should deploy its own domain smart contract DSC, which dictates the rules to be explicitly executed for their domain. In particular, all negotiated parameters between users and the SDN controller, for example, deposit fund, Internet usage, and current density level (see Table 3 for a complete list) must be recorded and processed in the smart contract. All setter/getter methods for “database” in Algorithms 1–4 can also be implemented in smart contracts for high integrity guarantee.

Aside from storing data, DSC must provide three essential methods for our system. First, DSC must have a deposit method, where users can allocate funds to be charged for their Internet usage. Calling to this method will freeze specified funds for future use. DSC also has a refund method, which allows users to unfreeze funds and return their deposits. Second, the controller can add proof of usage by hashing the FLOW-REMOVED message it receives from the switch. The controller uploads the hash proof to DSC to finalize operations within a timeslot. Third, the controller can withdraw the deposits from users after submitting the proof. DSC calculates the bills based on users’ upload/download Internet usage and the congestion level at the time.

#### 4.3.2. Registry Smart Contract

Since many deployed DSC can co-exist in our environment, we need a separate contract to track DSC. The registry smart contract RSC is owned and maintained by a trusted third party (e.g., government or shareholders) and is responsible for holding the contract address of deployed DSC. Those third parties should audit and authenticate DSC before they are approved. Once a DSC’s address is registered in RSC, we can safely trust this DSC.

#### 4.3.3. Token Smart Contract

The token smart contract TSC is implemented as an ERC20 token [23] and functions as the payment method in our system. The contract provides basic methods such as mint, burn, transfer, and allowance features. Because many ERC20 token contracts exist in the blockchain network, the admin must specify which kind of TSC currency is accepted in DSC by registering the TSC address in DSC.

Furthermore, for safety reasons, we slightly modify the logic in TSC from the original ERC20 token to add a minor authentication during the contract calls. The allowance(·) and transferFrom(·) methods can only be called by a trusted DSC, which can be verified through the isRegisteredDomain(·) method in the RSC.

### 4.4. Proof of Usage Design

The proof of usage is used to verify whether the SDN controller has processed the Internet usage correctly. The proof can be verified in two ways: from the OpenFlow messages and the recorded metadata in smart contracts, as shown in Table 3.

*Proof that users exist in a given domain*. At the beginning of each timeslot, all users that want to consume the bandwidth in a given domain must be authenticated (see Algorithm 2). In particular, the user must be registered in DSC and have enough tokens deposited for data transfer within a timeslot. Once authenticated, the controller sends the FLOW-MOD messages to the switches to forward the users’ traffic. Based on these explanations, we can guarantee that only registered users are processed in the domain.

*Proof that users have consumed given amounts of upload and download Internet usage*. At the end of each timeslot, the SDN controller records the users’ Internet usage based on the statistics from the FLOW-REMOVED messages (see Algorithm 3). In particular, the controller may receive a set of FLOW-REMOVED messages such as $Z=\{Z_{1},Z_{2},\ldots ,Z_{i}\}$. The controller then must hash them together such that $y=H(Z_{1}\|Z_{2}\|\ldots \|Z_{i})$. *i* is the number of FLOW-REMOVED messages within $T_{n}$. Finally, the controller uploads the hash proof *y* to DSC to finalize operations within a timeslot.

Once the proof is submitted, the controller can charge the Internet usage bill by withdrawing from the users’ deposit (see Algorithm 5). The bill calculation is performed on-chain for high integrity. During validations, a validator must check if the submitted proof *y* is valid by inspecting the original messages Z. For this reason, the SDN controller must prepare some physical storage to temporarily store received OpenFlow messages for auditing.

Additionally, the controller can also put the original PACKET-IN message into the mix when generating *y*. This way, we can provide even stronger reasoning for why such FLOW-MOD messages (and eventually FLOW-REMOVED messages) exist in the first place. To provide an even stronger integrity guarantee, a further modification can also be made to switch hardware such that all PACKET-IN and FLOW-REMOVED messages from the switches must be accompanied by their own signature and timestamp. This way, we can strongly prove that only the switch can generate such messages at the mentioned time.

### 4.5. Comparison with Previous Work

Table 4 summarizes the comparison between our work and the existing works. Similar to ours, References [3,9,10] also have client configuration settings to control how the clients behave on different congestion levels. In particular, they configure user applications with a delay-sensitive setting. Applications that are not urgent and can be delayed will be put on low priority by transmitting their data only when the system detects off-peak congestion levels. This configuration requires the ISP to know client information (e.g., the list of applications and the clients’ delay preferences per application). Meanwhile, our client configuration is performed on the network level in an application-agnostic way, where we limit user traffic from all applications based on the current congestion level. By managing this way, the ISP does not have to know any information about applications installed on user devices, providing better privacy settings for clients.

The RTP works of [9,10] focus on the mathematical modeling of the proposed system to show that the proposed dynamic pricing is effective compared to UBP. Technical aspects and prototype implementation from their proposal have not been proposed yet. Contrary to the mentioned proposals, we propose a complete design and implementation of RTP and pay more attention to the technical aspect and feasibility of the RTP system.

Finally, our proposal is the only one that offers integration of the RTP system with SDN and blockchain, where we can provide many features such as user authentication, deposit, refund, Internet usage storage, Internet bill charging, and auditing in a single platform. All of those features can be performed seamlessly and are automated, effectively reducing the opex costs of conventional RTP systems.

## 5. Experimental Results

**Figure 2:**
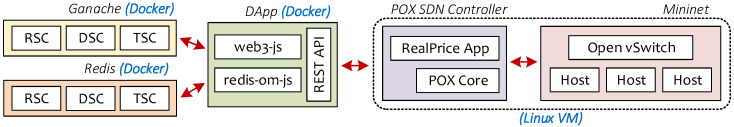
The testbed environment used for our evaluation. Mininet, POX SDN controller, and our SDN application reside in a single Linux virtual machine (VM). Meanwhile, the decentralized application (dapp) exists in a separate Docker container along with Ganache (for with-blockchain case) and Redis (for without-blockchain case).

We perform quantitative and qualitative analyses to show the usefulness of our proposal. The experiment is performed on hardware with the following specifications: Intel Core i7-10700K CPU @ 3.80 GHz and Samsung DIMM @ 2667MHz RAM. Figure 2 summarizes the testbed environment for our evaluation.

### 5.1. Off-Chain Performance Analysis

We analyze parts of our paper that do not relate to blockchain in this subsection, mainly regarding the feasibility of our proposal from the SDN side, which is implemented as an application in the POX SDN controller [24].

#### 5.1.1. Internet Bill Calculation Analysis

**Figure 3:**
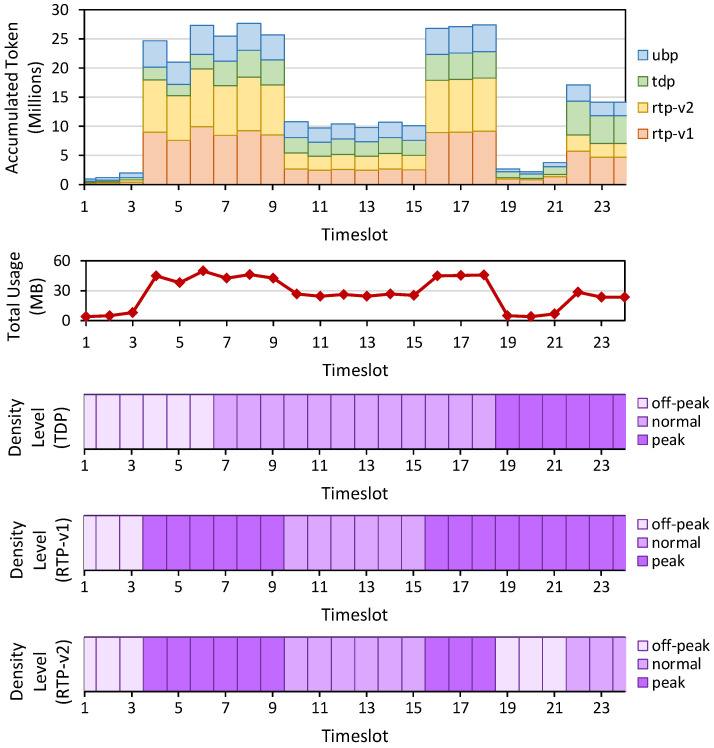
The experiment results for calculating the Internet bill (from (**top**) to (**bottom**)) showing the accumulated token and the byte usage per timeslot for UBP, TDP, RTP-v1, and RTP-v2, and the recorded density level for TDP, RTP-v1, and RTP-v2.

*Setup*: We first add a traffic density schedule in the system for the TDP scenario, with a 1:2:1 ratio of off-peak, normal, and peak distribution over 24 timeslots, as shown in Figure 3 (third figure from top). We then control four hosts (each has 10 Mbps bandwidth with about 40 Mbps total bandwidth domain) in Mininet to generate dummy traffic such that it will generate a traffic pattern as shown in Figure 3 (second figure from top). This traffic pattern does not follow the original TDP plan, which is intentional as we want to capture the flexibility of RTP to react to the current traffic.

The off-peak, normal, and peak pricing rates are set to 50, 100, and 200 token/KB. Based on the previously recorded byte usage and price information, we analyze the accumulated token that the system receives when they implement UBP, TDP, RTP-v1, and RTP-v2. Figure 3 (top) summarizes the accumulated token for each scenario, while the two bottom-most charts depict the analyzed density level for RTP-v1 and RTP-v2.

In RTP-v1 (proactive approach), the system executes **Rule 3** from the Density Tracker module by resetting/replacing the current density with a previously planned schedule from TDP. In other words, the network administrators perform a proactive approach by installing density levels based on their prediction results. The ISP may judge future density by observing previous traffic patterns’ history.

Meanwhile, in RTP-v2 (reactive approach), the system performs **Rule 3** by setting a default off-peak density at the beginning of each timeslot. This way, the system learns and detects the traffic congestion in the given timeslot and then applies the corresponding responses accordingly based on **Rule 1** and **Rule 2**.

*Results*: From our experiment, we confirm that the proposed RTP approach can adapt to the current traffic conditions by updating the initial plan from TDP to match the traffic (see two bottom-most charts in Figure 3). The system also generates more revenue when implemented in RTP than in TDP or UBP, as seen in Table 5. For UBP, we use the pricing rate of normal density, 100 token/KB. The RTP-v1 produces more tokens than the RTP-v2 because, at the last runs (from $T_{19}$ onward), the system does not apply the density level according to the congestion level but follows the original TDP plan. Recall that **Rule 2** prevents the density level from decreasing within a given timeslot, so the RTP-v2 applies peak density to the rest of the timeslot.

#### 5.1.2. User’s Internet Profile Analysis

**Figure 4:**
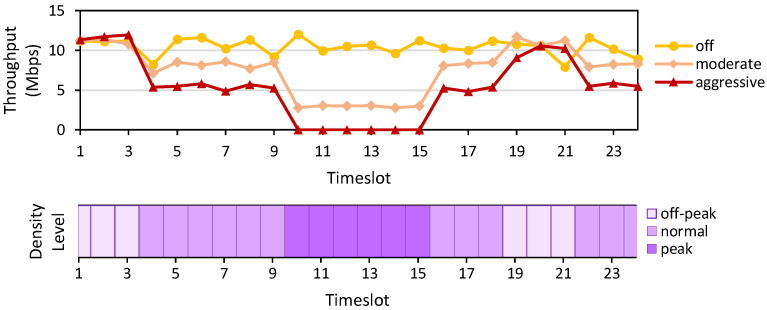
The experiment results for the Internet profile (from (**top**) to (**bottom**)) show the average throughput per timeslot using different Internet profiles and the recorded density level per timeslot.

*Setup*: One host in Mininet with a 10 Mbps bandwidth runs iperf [25] continuously to a server outside the domain. At the same time, other hosts in the same domain perform different tasks such that collaboratively they generate a congestion level pattern, as recorded in Figure 4 (bottom). Throughout this scenario, the host running the iperf is installed with three different Internet profiles, as previously described in Table 2. We measure the average throughput per timeslot for the iperf host, which is depicted in Figure 4 (top).

*Result*: Based on the displayed results, we confirm that our implemented SDN application can dynamically adjust the bandwidth of iperf host according to the currently installed Internet profile. In the case of an Aggressive profile, the app can drop the traffic when the peak congestion level is detected. The traffic is then resumed when the congestion dissipates.

### 5.2. On-Chain Performance Analysis

In this second part of the evaluation, we analyze parts of our proposal related to blockchain and smart contracts.

#### 5.2.1. Smart Contract Complexity Analysis

We analyze the contract sizes and the gas usage consumption per method to assess the complexity of our implemented smart contract.

*Setup*: We run a Ganache [26] network in a Docker container with the specification of 1 core of CPU and 1 GB of RAM. We use Truffle J.S. [27] to deploy our contracts to Ganache and measure the recorded byte sizes per contract and the gas usage per method. Table 6 and Table 7 summarize the result.

*Results of Byte Size*: All of our deployed contracts are within the byte size limit of 24 kilobytes (KiB), which is the upper bound limit to be deployed in the main the Ethereum network [28]. Thus, we can confirm that our contracts can be run in production cases. The Interface has no implemented methods, resulting in zero byte sizes.

*Results of Gas Consumption*: Our methods are also within the upper bound limit of 30 million gas in Ethereum network [29]. Therefore, we can also confirm that all implemented methods are executable in the production cases.

The resulting gas usage $g_{usage}$ and the block interval $b_{interval}$ have influences on the total number of transactions per second (TPS) that the blockchain network can process. Because of the gas limit $g_{limit}$, the higher the gas usage, the lesser transaction can be inserted into the block, resulting in overall TPS decreases. On the other hand, the faster we create blocks (lesser block interval value), the more transactions can be processed, increasing the TPS. Therefore, we can calculate the expected TPS per method using the following formula.

(1) $$tps=\left(g_{limit}/g_{usage}\right)/b_{interval}$$

We present the projected TPS per method in Table 7 if using Mainnet (13-s block interval [30]), Kovan (4-s block interval [31]), and Klaytn (1-s block interval [32]) blockchain network.

The initialization cases (Case I and II) are the most expensive operations since they include contract deployment. However, they are only performed once in a lifetime; hence, the higher gas usage and low throughput should be manageable. Similarly, the user setup (Case III) is also performed less frequently than others. Once users are registered, the number of calls to these methods will be reduced drastically.

On the other hand, Case IV is a collection of routine operations per timeslot, which users and SDN controllers will perform many times during a timeslot. Calls to these methods must then be processed as fast as possible. The given numbers in Case IV of Table 7 are specific for one user and recorded as a per-second value. Since we process Case IV for only one time per user per timeslot, the given low TPS value is actually still manageable. For example, if we set the timeslot as 1 hour, then we should multiply the raw TPS values by 3600× to get the approximation of the actual number of executions that can be made within a timeslot.

#### 5.2.2. Processing Delay Analysis

We measure the processing delay from our prototype to assess the overhead of performing tasks with and without blockchain.

*Setup*: We run a Ganache [26] network in a Docker container with the specification of 1 core of CPU and 1 GB of RAM. For a fair comparison, we also run Redis [33] with the same specification in a separate Docker container. The decentralized application (dapp) is also using a separate Docker container with 1 core of CPU and 1 GB of RAM (see Figure 2 for testbed layout). The dapp is implemented as REST API endpoints using Node JS. We ensure that the logic from the RSC, TSC, and DSC are all replicated similarly for our Redis cases. We then performed writable and read-only operations in our system 50 times and summarized the results in Table 8 and Table 9.

*Result*: Due to the high integrity of the blockchain process (i.e., signed transaction, consensus, and EVM stack), we can see a trade-off of higher processing delay by up to 21× compared to the conventional database (Redis). In particular, we can see an increase of 14.72× more delay (on average) for all writable operations and 6.74× for all read-only operations. The read-only methods are about 2× quicker than writable ones because they consist of more straightforward logic. Furthermore, read-only methods also do not modify the blockchain network’s state, so creating transactions is unnecessary. Therefore, this delay represents the raw query delay for data stored in EVM.

Note that despite Redis yielding lower delay, implementing our system in Redis does not have any meaningful benefits due to a lack of security and integrity. For example, the ERC20 token implemented as a database object is insecure and prone to tampering. Thus, the tokens should not have any economic value because no people will trust them. Finally, we also have to mention that this delay is recorded in our local testbed. Real-world latency may increase the delay further in production cases.

### 5.3. Security and Fairness Analysis

The followings are several design decisions made in this paper to guarantee the security and fairness of the proposal.

#### 5.3.1. Density Level Policy Analysis

This section analyzes the effectiveness of our density level policy (i.e., **Rules 1**–**3** in Section 4.2.5) when reacting to the simulated traffic pattern.

*Setup*: The IoT domain only contains two consumers; each is assigned about 10 Mbps bandwidth capacity. Therefore, the domain’s total bandwidth capacity is 20 Mbps. We use the density parameters (e.g., L, *j*) as specified previously in Algorithm 4. The user policy is also configured such that consumers send 100% of capacity during off-peak and normal. Meanwhile, consumers 1 and 2 send 10% and 50% of capacity during peak. The timeslot window is set as 12 min, and we receive PORT-STATISTIC messages every 1 min.

**Figure 5:**
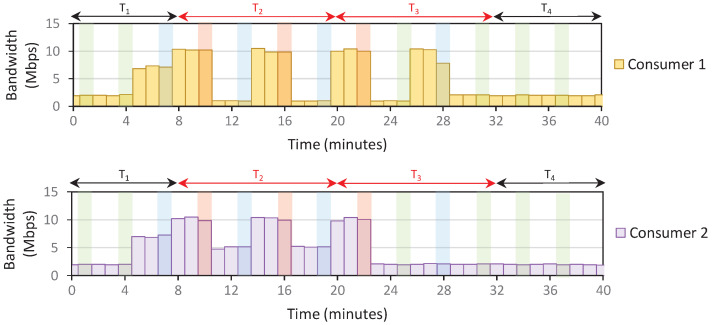
An example of a traffic pattern that only follows **Rule 1** (Scenario 1). Green, blue, and red bars point to density changes in off-peak, normal, and peak. The red color of $T_{n}$ indicates the point-of-interest timeslot, where we sent the 900 MB data.

**Figure 6:**
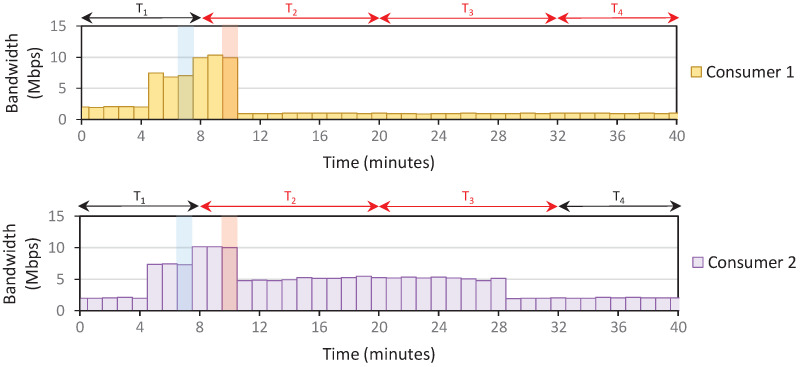
An example of a traffic pattern that only follows **Rules 1** and **2** (Scenario 2). Green, blue, and red bars point to density changes in off-peak, normal, and peak. The red color of $T_{n}$ indicates the point-of-interest timeslot, where we sent the 900 MB data.

**Figure 7:**
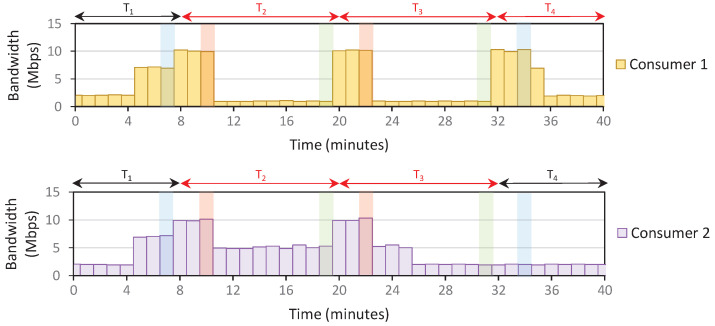
An example of a traffic pattern that follows **Rules 1**–**3** (Scenario 3). Green, blue, and red bars point to density changes in off-peak, normal, and peak. The red color of $T_{n}$ indicates the point-of-interest timeslot, where we sent the 900 MB data.

The consumers send arbitrary traffic throughout the scenario. However, starting at t=8 min, we assume that the consumer stops all running process and only send 900 MB of data in the domain. This should result in 10 Mbps data transfers over 12 min (if no data restriction is applied). After all 900 MB data is transferred, consumers resume the previous activity, sending random traffic at about 2 Mbps data rate. We performed three scenarios: the system only applies **Rule 1** (Scenario 1), **Rules 1**–**2** (Scenario 2), and **Rules 1**–**3** (Scenario 3). The traffic patterns from those scenarios are depicted in Figure 5, Figure 6 and Figure 7. We assume that the density starts with an off-peak level at $T_{1}$.

*Results*: The first peak density is detected at t=10, and consumers react by reducing traffic. In Scenario 1, this traffic reduction means that the system recalculates the current rate and detects a normal density at t=13. Therefore, the consumers increase the traffic again to overload the network at t=16. These back-and-forth density level changes happen continuously until all the 900 MB of data are fully transferred, making it confusing for consumers to determine the current density level at a given timeslot.

On the other hand, the density level is unchanged after the first peak in Scenario 2. However, because there is no policy to reduce the density level, the peak status is carried over to the subsequent timeslots, resulting in a stagnant level. Scenario 3 fixes the problems in Scenario 2, where the density resets at the beginning of each timeslot. This allows the system to recalculate the density briefly and adjust the density more accurately, especially when the traffic congestion is reduced, such as in $T_{4}$ of Figure 7.

*Lesson Learned*: By complying with all **Rules 1**–**3**, we solve the trade-off between ensuring that the system can accurately detect the dynamics of traffic patterns while providing clear pointers for consumers regarding the current density level in a given timeslot. Furthermore, consumers willing to take the risk (and pay more bills) by transmitting more bytes during peak hours will receive benefits such as a faster data transfer completion rate than saving-oriented consumers. Figure 7 shows that consumer 2 completes about 37% faster than consumer 1.

Note that the system takes 3 min (or 25% of the timeslot window) to determine the density level. This is performed for the sake of easy explanations, where everything is calculated in minutes. In the production case, the detection can be made in seconds instead of minutes, so it does not take much time to detect the density level.

#### 5.3.2. Token and User Interaction Analysis

*Only registered and well-funded clients can access the Internet*. Because we design the Internet bill in a pre-paid fashion, users must have token deposits in the system before accessing the Internet. In particular, users must prepare funds for at least one timeslot. Otherwise, the controller will reject users’ access.

*An SDN controller can withdraw the deposited funds from users only after submitting the proof*. This policy is to ensure that the SDN controller has some proofs that they have performed the usage byte measurement correctly.

*All critical negotiations must be made on-chain*. The token deposit, refund, and withdrawal must be made through DSC and TSC. The users’ byte usage and proof of usage must also be uploaded to the blockchain. Therefore, all essential parameters can be securely audited when needed.

## 6. Discussion and Future Works

Our paper can be extended in five ways, mainly regarding privacy-preserving, fault-tolerance, integrity, user identification, and pricing mechanism.

First, the use of blockchain can be a double-edged sword. On the one hand, blockchain transparency helps audit the system securely. On the other hand, it also means that user privacy is at risk. While blockchain users’ identity is masked with anonymous blockchain addresses, if adversaries can reveal the true identity behind those addresses, then users’ Internet usage history can be leaked. Adversaries can also spot rich/poor domains by observing the total deposited funds in the contract. This can be used as an avenue to pick their criminal target. A possible solution is limiting access to the stored ledger data using a permissioned blockchain. Other solutions include improving the privacy-preserving aspect of the blockchain network itself.

Second, the system may lose ongoing Internet usage metrics when switch failures happen. Recall that the edge switch records users’ Internet usage through the inserted flow rules. Unless there is a method to save/cache the state of the Flow Table in the switch locally, this information might be deleted when the switch fails, and the controller will not obtain an accurate usage metric during this period.

Third, further modifications to the SDN switch and controller hardware can be made to provide a stronger integrity guarantee. In particular, typical SDN switches do not have trustful timestamp resources and a robust cryptography module. Putting a Trusted Execution Environment (TEE) module can boost the Internet usage proof generated from switches. Furthermore, the SDN controller needs additional storage to temporarily save OpenFlow messages submitted by switches for auditing.

Fourth, our proposed system relies heavily on the MAC address for user identification and usage monitoring. However, this paper only provides minimal user authentication setup required to perform RTP. Because of this, performing MAC spoofing attacks is still possible in the system, and further advanced MAC spoofing detection, such as in [34,35], must be integrated into the system. Furthermore, using a NAT gateway or similar proxy systems is not possible inside the domain because they will translate users’ MAC addresses into proxies MAC addresses. The system then cannot detect users sitting behind proxy servers unless the servers share some information with the SDN controller. Therefore, the developers are encouraged to put the SDN switch as close to the edge as possible.

Finally, the optimal formula to define dynamic pricing has not been proposed yet. This step requires real user trial and feedback to determine the best pricing scheme for consumers and ISP.

## 7. Conclusions

This paper proposed a novel real-time pricing mechanism that leveraged SDN and blockchain. We tracked the congestion level in an SDN edge domain and adjusted the pricing rate dynamically. The controller generated bills for users through smart contracts based on their Internet consumption and the congestion level at that time. Furthermore, to prevent overcharging, users could create an Internet profile, which limits how much bandwidth is to be delivered across various congestion levels.

Through our prototype evaluation, we have shown that the system is feasible and can perform all of the proposed workflows correctly. The smart contract byte sizes and gas costs to execute all smart contract methods were within the boundary of Ethereum’s main network limit. Integrating the POX SDN controller and Ethereum blockchain to track users’ Internet usage and charge the bill also worked seamlessly.

More importantly, we showed that by using our approach, the ISP could get up to 72.91% more profit compared to the existing approach. This highlighted the efficiency of our system in detecting real-time network congestion and adjusting the pricing rate accordingly. The use of blockchain added, on average, 14.72× more delay on writable operations and 6.74× more on read-only operations compared to using a conventional database such as Redis DB. This showed the trade-off developers need to consider when deploying our system for production cases such that the high integrity guarantee from blockchain comes with a longer processing delay. Thus, a strategy to minimize the blockchain delay includes a hybrid approach, where we offload some parts of our system that do not need a high-integrity guarantee to the Redis DB.

Finally, future works from our paper include solving the privacy issues, enhancing switch failure robustness, adding a lightweight module to the switch’s hardware for generating secure cryptography and timestamping, and alleviating the MAC spoofing attacks. Real user trials and feedback through real-hardware implementation can also be performed in the future to test the usability and practicality of the proposed RTP system.

## Figures and Tables

**Table 1:** List of notations used in this paper.

Notation	Description
RSC/TSC/DSC	Registry/Token/Domain smart contract
u/c/s	Users (Consumers)/SDN controllers/SDN switches
R/U/C	A list of registered Domain contracts/consumers/SDN controllers
$\mu_{src}/\mu_{dst}$	Source/Destination MAC address
$\gamma_{src}/\gamma_{dst}$	Source/Destination IP address
$\theta_{src}/\theta_{dst}$	Source/Destination TCP or UDP port number
$\omega /\omega^{cap}$	Bandwidth/Total bandwidth capacity
$M/\psi_{u}$	Flow rules matches/Internet profile (Queue identifier)
L/Q/J	A list of congestion level/bandwidth thresholds/counters
$T/t/t_{now}$	Timeslot/Timestamp/Current timestamp
α/p/d	Blockchain address/Switch’s port/Density (Congestion) level
δ/β/ρ	Deposit/Balances/Price rate
B/P	Byte count/Packet count
$B^{up}/B^{down}$	Upload byte usage/Download byte usage
y/Y/b	Proof of Internet usage/A list of usage proofs/Internet usage bills
H(·)	Hash payload using KECCAK-256 or SHA-256 hash function
X‖Y	A concatenation of *X* and *Y*

**Table 2:** An example of users’ Internet profile stating how much bandwidth they want to utilize during various congestion levels.

Type	Off-Peak	Normal	Peak
Off	100%	100%	100%
Moderate	100%	75%	25%
Aggressive	100%	50%	0%

**Table 3:** List of metadata stored on-chain.

Category	Contract	Metadata	Type of Proof
Identity	RSC,DSC	$\alpha_{DSC},\alpha_{u},\alpha_{c}$	Verify that such entity exist
Location	DSC	$\left\{\alpha_{u},\alpha_{DSC}\right\}$	A mapping that shows users are inside DSC
Bandwidth	DSC	$\omega_{u},\omega_{domain},B_{u}$	Verify that the user Internet usage is valid
Pricing	DSC,TSC	$B_{u},\rho_{n},d_{n}$	Prove that the bill is calculated correctly at $T_{n}$

**Table 4:** Feature comparisons with existing RTP works.

Reference	Client Config	Proto.	SDN.	Block.	Auth.	Bill.	Proof.
Gu et al. [9]	Delay-based	–	Yes	–	–	–	–
Gu et al. [10]	Delay-based	–	Yes	–	–	–	–
Ha et al. [3]	Delay-based	Yes	–	–	Yes	Yes	–
Ours	QoS-based	Yes	Yes	Yes	Yes	Yes	Yes

*Proto.*: Prototype implementation. *SDN.*: Software-defined networking. *Block.*: Blockchain. *Auth.*: User authentication. *Bill.*: Internet bill charge. *Proof.*: Proof of Internet usage.

**Table 5:** Total accumulated tokens from our experiment in Figure 3.

	RTP-v1	RTP-v2	TDP	UBP
Total Accumulated Tokens	114,442,792	104,495,907	67,856,817	66,185,036
% Increase to UBP	72.91%	57.88%	2.53%	0.00%

**Table 6:** The sizes of all deployed smart contracts. We assume the contract size limit is 24 KiB.

Contract Name	Type	Used For	Size (KiB)	% Limit
Registry	Contract	RSC	0.82	3.42
IRegistry	Interface	RSC	0.00	0.00
ERC20	Contract	TSC	5.34	22.25
IERC20	Interface	TSC	0.00	0.00
Region	Contract	DSC	9.35	38.96
IRegion	Interface	DSC	0.00	0.00
SafeMath	Library	All	0.08	0.33

**Table 7:** List of writable smart contract methods and their gas consumption. We assume the block limit of 30 million gas. The estimated throughput in transactions per second (TPS) is calculated based on the block interval in Mainnet, Kovan, and Klaytn networks.

Description	C*	Contract	Method	Gas Usage	% Limit	Throughput (TPS)		
						Mainnet	Kovan	Klaytn
*Case I: Registry Initialization*								
Deploying SafeMath library contract	*a*	SafeMath	deploy	71,933	0.24	32.08	104.26	417.05
Deploying Registry contract	*a*	Registry	deploy	255,184	0.85	9.04	29.39	117.56
Deploying Token contract	*a*	ERC20	deploy	1,320,662	4.40	1.75	5.68	22.72
			*Total*	1,647,779	5.49	1.40	4.55	18.21
*Case II: Domain Initialization*								
Deploying Domain contract	*a*	Region	deploy	2,230,667	7.44	1.03	3.36	13.45
Linking Domain contract to Registry	*a*	Registry	addRegion	44,606	0.15	51.74	168.14	672.56
Updating pricing rate in domain	*a*	Region	setPricingRate	41,840	0.14	55.16	179.25	717.02
Updating timeslot window	*a*	Region	setWindow	29,315	0.10	78.72	255.84	1023.37
			*Total*	2,346,428	7.83	0.98	3.20	12.79
*Case III: User Setup*								
Registering user	*u*	Region	addUser	44,688	0.15	51.64	167.83	671.32
Updating Internet profile	*u*	Region	setProfile	30,962	0.10	74.53	242.23	968.93
Updating usable bandwidth	*u*	Region	setBandwidth	45,096	0.15	51.17	166.31	665.25
Minting tokens for users	*a*	ERC20	mint	66,265	0.22	34.83	113.18	452.73
Depositing tokens for Internet usage	*u*	Region	deposit	52,346	0.17	44.09	143.28	573.11
			*Total*	239,357	0.79	9.64	31.33	125.34
*Case IV: Day-to-Day Operational per Timeslot*								
Updating current congestion level	*c*	Region	setDensity	45,643	0.15	50.56	164.32	657.27
Adding download byte usage	*c*	Region	addDownload	47,685	0.16	48.39	157.28	629.13
Adding upload byte usage	*c*	Region	addUpload	47,729	0.16	48.35	157.14	628.55
Adding hashes of FLOW-REMOVED	*c*	Region	addProof	46,984	0.16	49.12	159.63	638.52
Withdrawing the deposited tokens	*c*	Region	withdraw	57,066	0.19	40.44	131.43	525.71
Taking back deposited tokens	*u*	Region	refund	25,918	0.09	89.04	289.37	1157.50
			*Total*	271,025	0.91	8.51	27.67	110.69
*Case V: Others*								
Token transfers between accounts	*u*	ERC20	transfer	51,967	0.17	44.41	144.32	577.29
Burning tokens	*u*	ERC20	burn	37,256	0.12	61.94	201.31	805.24

*Callers* (C*). *a*: Admin. *u*: User. *c*: SDN Controller.

**Table 8:** Processing delay (in milliseconds) for writable operations in RealPrice when implemented as non-blockchain (Redis DB) and blockchain (Ganache) approaches.

Description	Method	Redis DB		Ethereum (Ganache)		
		Average	STD	Average	STD	Gap to Redis
Set timeslot window	setWindow	1.71	0.46	30.34	3.29	17.75×
Update pricing rate	setPrice	1.69	0.46	35.58	5.17	21.11×
Add new user	addUser	2.19	0.60	27.51	3.46	12.56×
Set Internet profile	setProfile	1.89	0.39	27.63	1.80	14.63×
Set user bandwidth	setBandwidth	1.94	0.43	27.66	2.97	14.29×
Update congestion	setDensity	2.24	0.73	32.29	3.25	14.43×
Add upload usage	addUpload	2.12	0.54	27.91	1.80	13.18×
Add download usage	addDownload	2.24	0.57	27.55	3.17	12.28×
Add hash proof usage	addProof	2.01	0.40	26.96	1.79	13.39×
Deposit funds	deposit	2.05	0.49	28.63	2.18	13.95×
Refund deposited fund	refund	1.76	0.46	30.69	4.52	17.46×
Withdraw bill	withdraw	3.23	0.52	37.47	2.34	11.59×

**Table 9:** Processing delay (in milliseconds) for read-only operations in RealPrice when implemented as non-blockchain (Redis DB) and blockchain (Ganache) approaches.

Description	Method	Redis DB		Ethereum (Ganache)		
		Average	STD	Average	STD	Gap to Redis
Get timeslot window	getWindow	1.44	0.31	10.63	1.67	7.40×
Get pricing rate info	getPrice	1.46	0.41	11.93	2.27	8.17×
Get user information	getUser	1.89	0.58	10.93	1.39	5.79×
Get bandwidth usage info	getUsage	1.76	0.55	12.48	1.55	7.08×
Authenticate user	isUserValid	2.86	0.63	15.14	1.77	5.29×

## Data Availability

Not applicable.

## References

[1] Odlyzko A., Arnaud B.S., Stallman E., Weinberg M. (2012). Know your limits: Considering the role of data caps and usage based billing in internet access service. Public Knowl..

[2] Jiang L., Parekh S., Walrand J. Time-dependent network pricing and bandwidth trading. Proceedings of the NOMS Workshops 2008-IEEE Network Operations and Management Symposium Workshops.

[3] Ha S., Sen S., Joe-Wong C., Im Y., Chiang M. TUBE: Time-dependent pricing for mobile data. Proceedings of the ACM SIGCOMM 2012 Conference on Applications, Technologies, Architectures, and Protocols for Computer Communication.

[4] Ali Y., Haque A., Bitar B. Towards the Development of a Novel Service Cost Modeling: An ISP Perspective. Proceedings of the 2020 International Symposium on Networks, Computers and Communications (ISNCC).

[5] Arkko J. (2020). The influence of internet architecture on centralised versus distributed internet services. J. Cyber Policy.

[6] Thaichon P., Quach T.N. (2015). The relationship between service quality, satisfaction, trust, value, commitment and loyalty of Internet service providers’ customers. J. Glob. Sch. Mark. Sci..

[7] McKeown N., Anderson T., Balakrishnan H., Parulkar G., Peterson L., Rexford J., Shenker S., Turner J. (2008). OpenFlow: Enabling innovation in campus networks. ACM SIGCOMM Comput. Commun. Rev..

[8] Nakamoto S. (2008). Bitcoin: A peer-to-peer electronic cash system. Decent. Bus. Rev..

[9] Gu B., Feng J., Zhou Z., Guizani M. Time-dependent pricing for on-demand bandwidth slicing in software defined networks. Proceedings of the 2018 14th International Wireless Communications & Mobile Computing Conference (IWCMC).

[10] Gu B., Dong M., Zhang C., Liu Z., Tanaka Y. Real-time pricing for on-demand bandwidth reservation in SDN-enabled networks. Proceedings of the 2017 14th IEEE Annual Consumer Communications & Networking Conference (CCNC).

[11] Nevo A., Turner J.L., Williams J.W. (2016). Usage-based pricing and demand for residential broadband. Econometrica.

[12] Sen S., Joe-Wong C., Ha S., Bawa J., Chiang M. When the price is right: Enabling time-dependent pricing of broadband data. Proceedings of the SIGCHI Conference on Human Factors in Computing Systems.

[13] Haxhibeqiri J., Isolani P.H., Marquez-Barja J.M., Moerman I., Hoebeke J. (2020). In-band network monitoring technique to support SDN-based wireless networks. IEEE Trans. Netw. Serv. Manag..

[14] Yao H., Mai T., Xu X., Zhang P., Li M., Liu Y. (2018). NetworkAI: An intelligent network architecture for self-learning control strategies in software defined networks. IEEE Internet Things J..

[15] Guan B., Shen S.H. FlowSpy: An efficient network monitoring framework using P4 in software-defined networks. Proceedings of the 2019 IEEE 90th Vehicular Technology Conference (VTC2019-Fall).

[16] Castro M., Liskov B. Practical byzantine fault tolerance. Proceedings of the 3rd Symposium on Operating Systems Design and Implementation.

[17] Buterin V. (2014). A next-generation smart contract and decentralized application platform. White Pap..

[18] Queiroz M.M., Telles R., Bonilla S.H. (2020). Blockchain and supply chain management integration: A systematic review of the literature. Supply Chain. Manag. Int. J..

[19] Attaran M. (2022). Blockchain technology in healthcare: Challenges and opportunities. Int. J. Healthc. Manag..

[20] Ouyang L., Yuan Y., Wang F.Y. (2020). Learning Markets: An AI Collaboration Framework Based on Blockchain and Smart Contracts. IEEE Internet Things J..

[21] Li M., Weng J., Yang A., Lu W., Zhang Y., Hou L., Liu J.N., Xiang Y., Deng R.H. (2018). CrowdBC: A blockchain-based decentralized framework for crowdsourcing. IEEE Trans. Parallel Distrib. Syst..

[22] Gül Ö.M. (2022). Blockchain-enabled Internet of Things (IoTs) platforms for vehicle sensing and transportation monitoring. Machine Learning, Blockchain Technologies and Big Data Analytics for IoTs: Methods, Technologies and Applications.

[23] Vogelsteller F., Buterin V. EIP-20: Token Standard. https://eips.ethereum.org/EIPS/eip-20.

[24] McCauley POX Manual Current Documentation. https://noxrepo.github.io/pox-doc/html/.

[25] Dugan J., Elliott S., Mah B.A., Poskanzer J., Prabhu K. iPerf—The Ultimate Speed Test Tool for TCP, UDP and SCTP. https://iperf.fr/iperf-doc.php.

[26] Truffle Suite Ganache: One Click Blockchain. https://trufflesuite.com/ganache/.

[27] Truffle Suite Truffle: Smart Contracts Made Sweeter. https://trufflesuite.com/truffle/.

[28] Mudge N. Ethereum’s Maximum Contract Size Limit is Solved with the Diamond Standard. https://dev.to/mudgen/ethereum-s-maximum-contract-size-limit-is-solved-with-the-diamond-standard-2189.

[29] Ethereum.org Gas and Fees. https://ethereum.org/en/developers/docs/gas/.

[30] Etherscan Ethereum Average Block Time Chart. https://etherscan.io/chart/blocktime.

[31] Etherscan Kovan Testnet Explorer. https://kovan.etherscan.io/.

[32] Klaytn Klaytn Overview. https://docs.klaytn.foundation/klaytn.

[33] Manchale A. (2021). Tug Grall on Redis. IEEE Softw..

[34] Lin T., Kang J.M., Bannazadeh H., Leon-Garcia A. Enabling SDN Applications on Software-Defined Infrastructure. Proceedings of the 2014 IEEE Network Operations and Management Symposium (NOMS).

[35] Tao K., Li J., Sampalli S. Detection of spoofed MAC addresses in 802.11 wireless networks. Proceedings of the International Conference on E-Business and Telecommunications.

